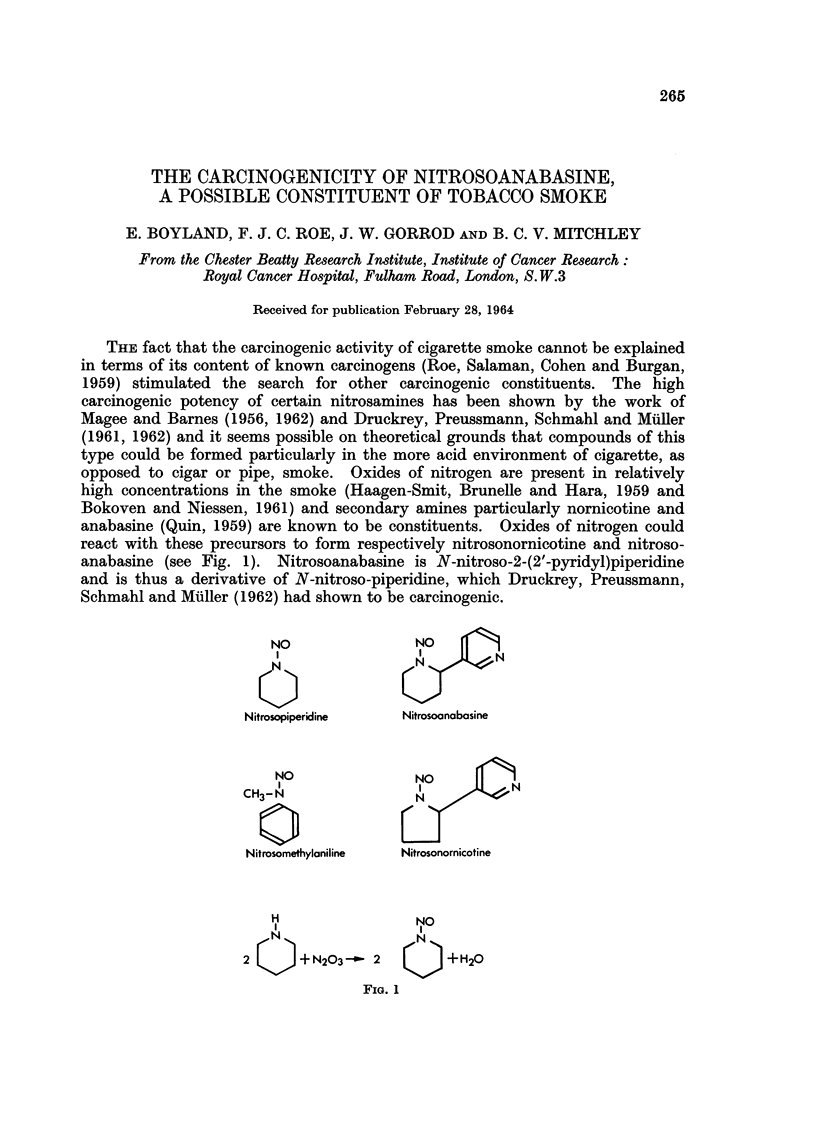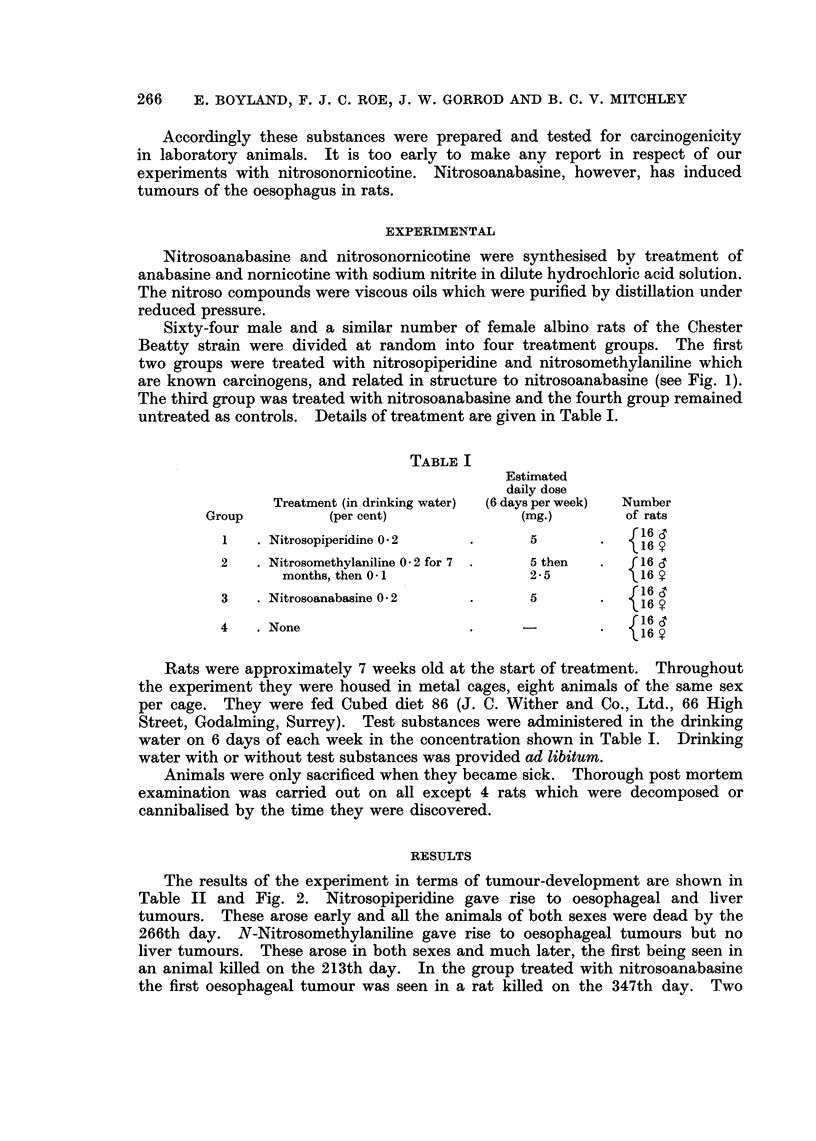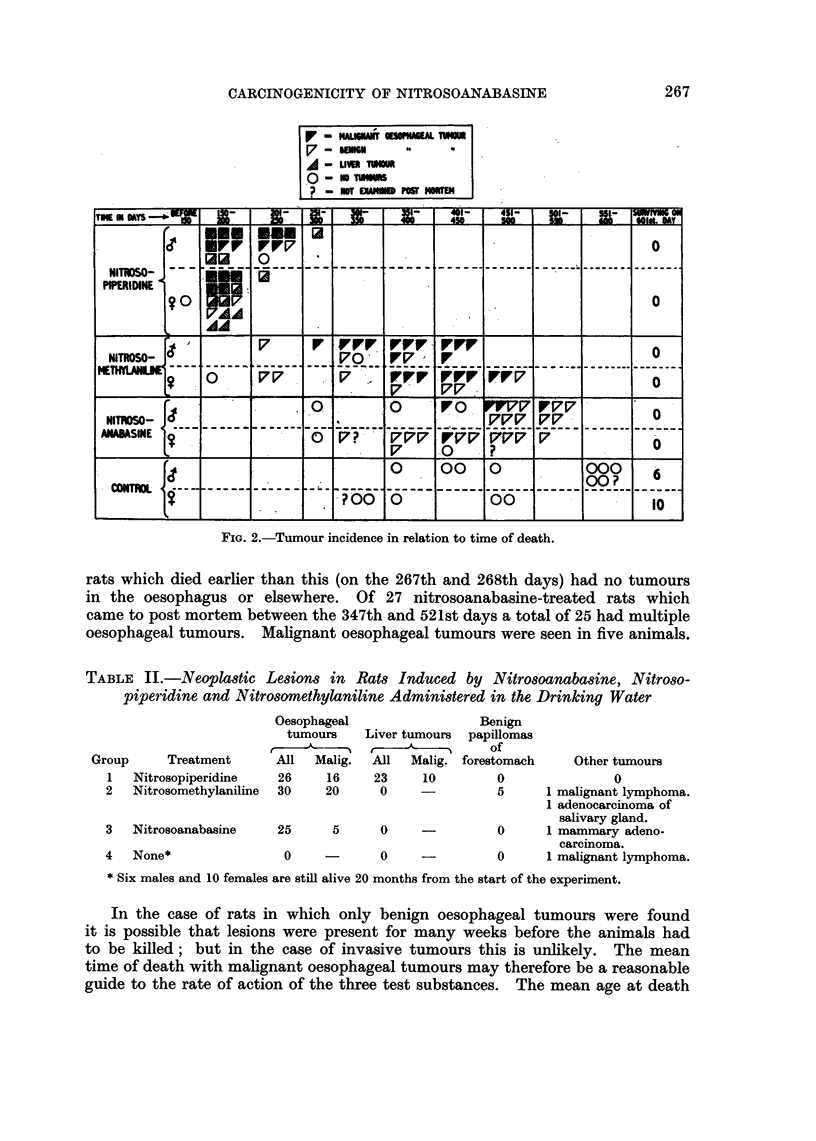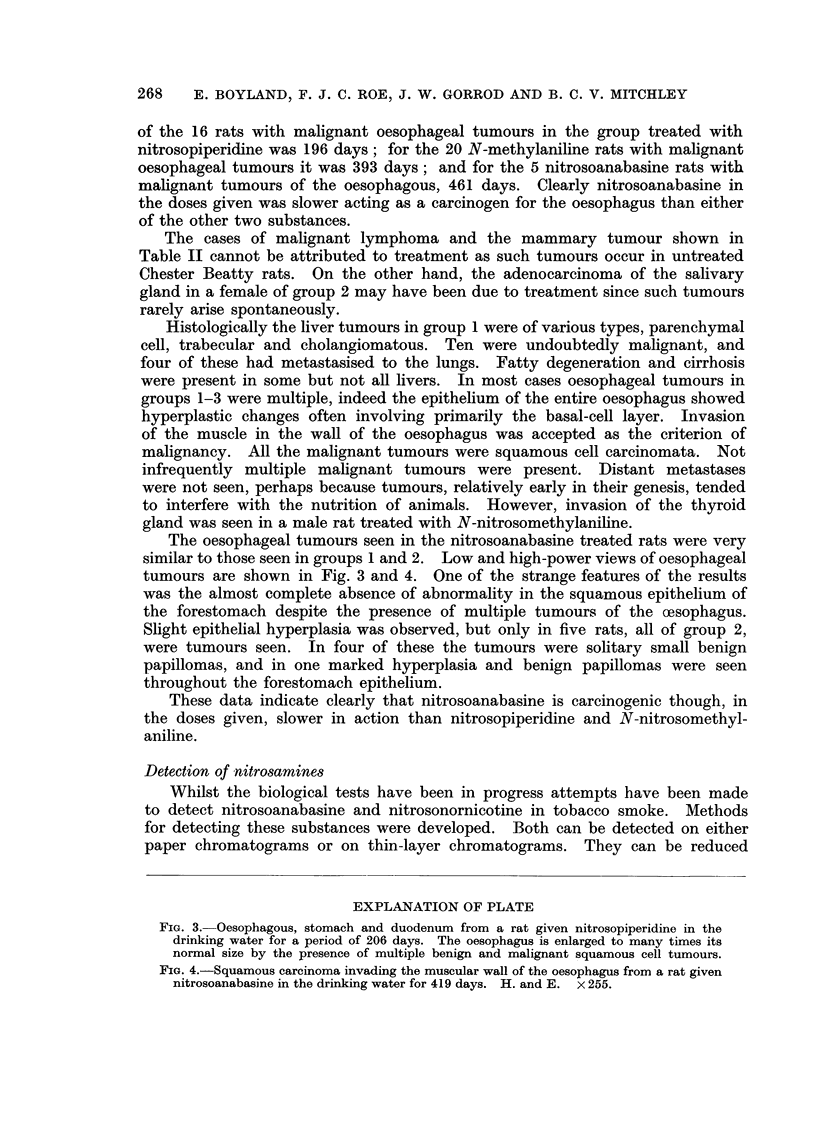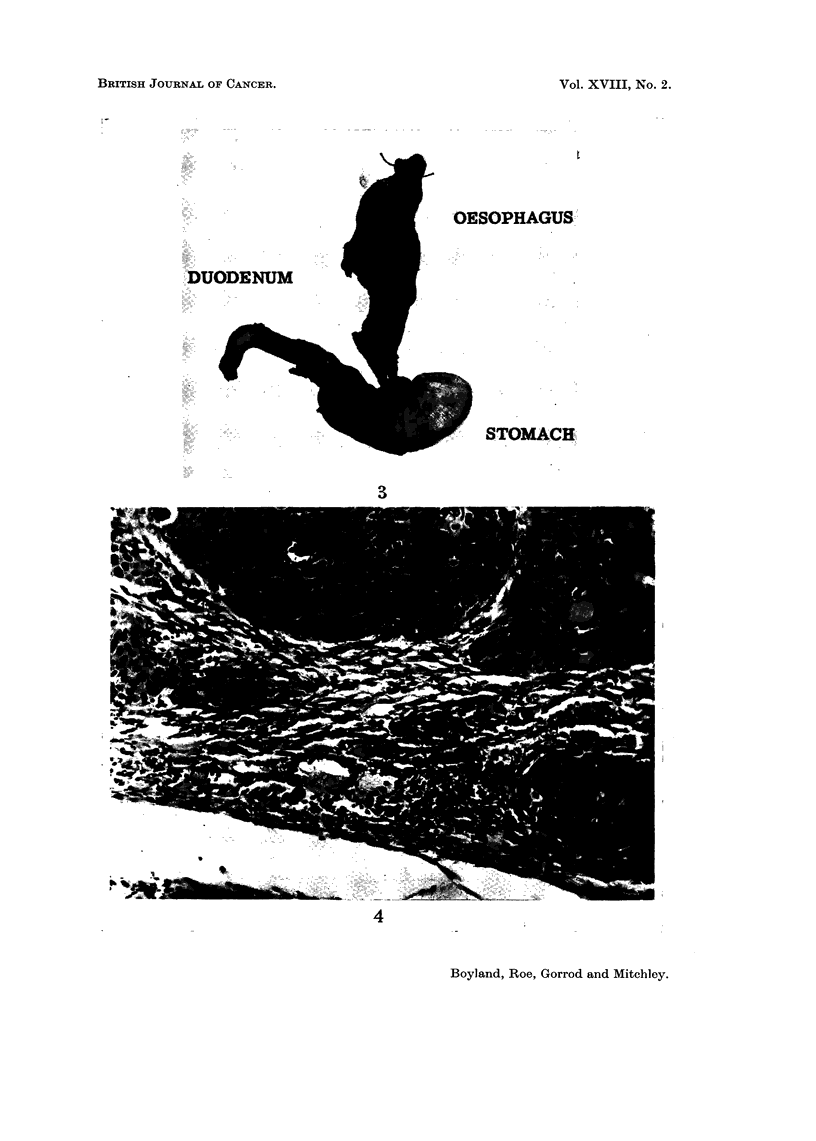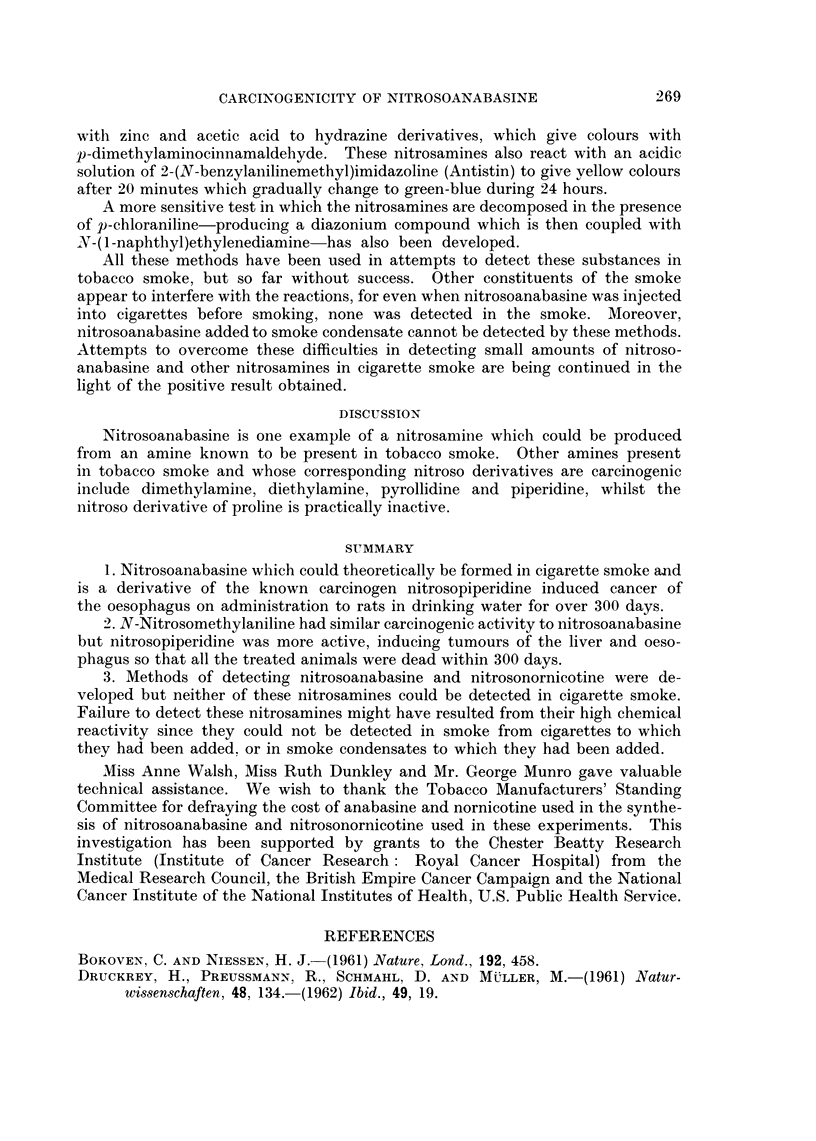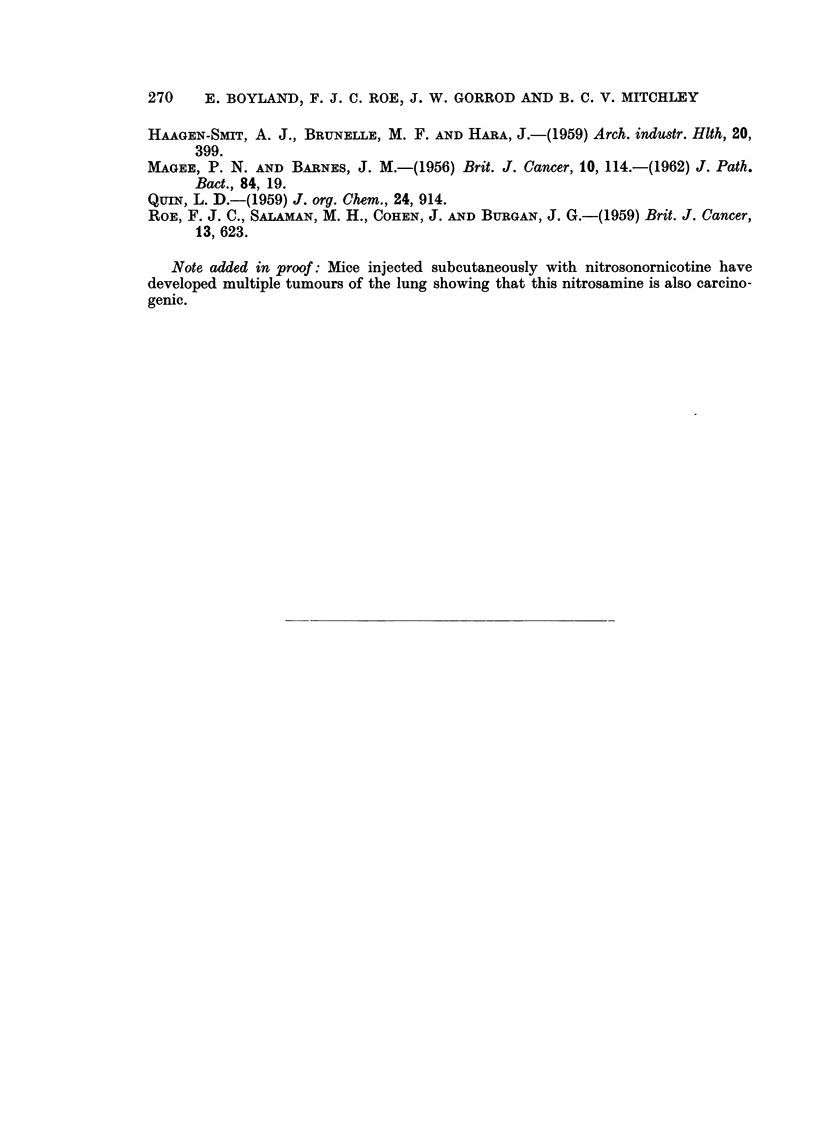# The Carcinogenicity of Nitrosoanabasine, a Possible Constituent of Tobacco Smoke

**DOI:** 10.1038/bjc.1964.31

**Published:** 1964-06

**Authors:** E. Boyland, F. J. C. Roe, J. W. Gorrod, B. C. V. Mitchley

## Abstract

**Images:**


### THE CARCINOGENICITY OF NITROSOANABASINE, A POSSIBLE CONSTITUENT OF TOBACCO SMOKE

E. BOYLAND, F. J. C. ROE, J. W. GORROD AND B. C. V. MITCHLEY

*From the Chester Beatty Research Institute, Institute of Cancer Research : Royal Cancer Hospital, Fulham Road, London, S.W.3*

Received for publication February 28, 1964

THE fact that the carcinogenic activity of cigarette smoke cannot be explained in terms of its content of known carcinogens (Roe, Salaman, Cohen and Burgan, 1959) stimulated the search for other carcinogenic constituents. The high carcinogenic potency of certain nitrosamines has been shown by the work of Magee and Barnes (1956, 1962) and Druckrey, Preussmann, Schmahl and Müller (1961, 1962) and it seems possible on theoretical grounds that compounds of this type could be formed particularly in the more acid environment of cigarette, as opposed to cigar or pipe, smoke. Oxides of nitrogen are present in relatively high concentrations in the smoke (Haagen-Smit, Brunelle and Hara, 1959 and Bokoven and Niessen, 1961) and secondary amines particularly nornicotine and anabasine (Quin, 1959) are known to be constituents. Oxides of nitrogen could react with these precursors to form respectively nitrosonornicotine and nitroso-anabasine (see Fig. 1). Nitrosoanabasine is *N*-nitroso-2-(2′-pyridyl)piperidine and is thus a derivative of *N*-nitroso-piperidine, which Druckrey, Preussmann, Schmahl and Müller (1962) had shown to be carcinogenic.

FIG. 1

Accordingly these substances were prepared and tested for carcinogenicity in laboratory animals. It is too early to make any report in respect of our experiments with nitrosonornicotine. Nitrosoanabasine, however, has induced tumours of the oesophagus in rats.

### EXPERIMENTAL

Nitrosoanabasine and nitrosonornicotine were synthesised by treatment of anabasine and nornicotine with sodium nitrite in dilute hydrochloric acid solution. The nitroso compounds were viscous oils which were purified by distillation under reduced pressure.

Sixty-four male and a similar number of female albino rats of the Chester Beatty strain were divided at random into four treatment groups. The first two groups were treated with nitrosopiperidine and nitrosomethylaniline which are known carcinogens, and related in structure to nitrosoanabasine (see Fig. 1). The third group was treated with nitrosoanabasine and the fourth group remained untreated as controls. Details of treatment are given in Table I.

TABLE I

| Group | Treatment (in drinking water) (per cent) | Estimated daily dose (6 days per week) (mg.) | Number of rats |
|---|---|---|---|
| 1 | Nitrosopiperidine 0·2 | 5 | 16 ♂ 16 ♀ |
| 2 | Nitrosomethylaniline 0·2 for 7 months, then 0·1 | 5 then 2·5 | 16 ♂ 16 ♀ |
| 3 | Nitrosoanabasine 0·2 | 5 | 16 ♂ 16 ♀ |
| 4 | None | — | 16 ♂ 16 ♀ |

Rats were approximately 7 weeks old at the start of treatment. Throughout the experiment they were housed in metal cages, eight animals of the same sex per cage. They were fed Cubed diet 86 (J. C. Wither and Co., Ltd., 66 High Street, Godalming, Surrey). Test substances were administered in the drinking water on 6 days of each week in the concentration shown in Table I. Drinking water with or without test substances was provided *ad libitum.*

Animals were only sacrificed when they became sick. Thorough post mortem examination was carried out on all except 4 rats which were decomposed or cannibalised by the time they were discovered.

### RESULTS

The results of the experiment in terms of tumour-development are shown in Table II and Fig. 2. Nitrosopiperidine gave rise to oesophageal and liver tumours. These arose early and all the animals of both sexes were dead by the 266th day. *N*-Nitrosomethylaniline gave rise to oesophageal tumours but no liver tumours. These arose in both sexes and much later, the first being seen in an animal killed on the 213th day. In the group treated with nitrosoanabasine the first oesophageal tumour was seen in a rat killed on the 347th day. Two

FIG. 2.—Tumour incidence in relation to time of death.

rats which died earlier than this (on the 267th and 268th days) had no tumours in the oesophagus or elsewhere. Of 27 nitrosoanabasine-treated rats which came to post mortem between the 347th and 521st days a total of 25 had multiple oesophageal tumours. Malignant oesophageal tumours were seen in five animals.

TABLE II.—*Neoplastic Lesions in Rats Induced by Nitrosoanabasine, Nitrosopiperidine and Nitrosomethylaniline Administered in the Drinking Water*

| Group | Treatment | Oesophageal tumours | | Liver tumours | | Benign papillomas of forestomach | Other tumours |
|---|---|---|---|---|---|---|---|
| | | All | Malig. | All | Malig. | | |
| 1 | Nitrosopiperidine | 26 | 16 | 23 | 10 | 0 | 0 |
| 2 | Nitrosomethylaniline | 30 | 20 | 0 | — | 5 | 1 malignant lymphoma. 1 adenocarcinoma of salivary gland. |
| 3 | Nitrosoanabasine | 25 | 5 | 0 | — | 0 | 1 mammary adeno-carcinoma. |
| 4 | None* | 0 | — | 0 | — | 0 | 1 malignant lymphoma. |

* Six males and 10 females are still alive 20 months from the start of the experiment.

In the case of rats in which only benign oesophageal tumours were found it is possible that lesions were present for many weeks before the animals had to be killed; but in the case of invasive tumours this is unlikely. The mean time of death with malignant oesophageal tumours may therefore be a reasonable guide to the rate of action of the three test substances. The mean age at death of the 16 rats with malignant oesophageal tumours in the group treated with nitrosopiperidine was 196 days ; for the 20 *N*-methylaniline rats with malignant oesophageal tumours it was 393 days ; and for the 5 nitrosoanabasine rats with malignant tumours of the oesophagous, 461 days. Clearly nitrosoanabasine in the doses given was slower acting as a carcinogen for the oesophagus than either of the other two substances.

The cases of malignant lymphoma and the mammary tumour shown in Table II cannot be attributed to treatment as such tumours occur in untreated Chester Beatty rats. On the other hand, the adenocarcinoma of the salivary gland in a female of group 2 may have been due to treatment since such tumours rarely arise spontaneously.

Histologically the liver tumours in group 1 were of various types, parenchymal cell, trabecular and cholangiomatous. Ten were undoubtedly malignant, and four of these had metastasised to the lungs. Fatty degeneration and cirrhosis were present in some but not all livers. In most cases oesophageal tumours in groups 1–3 were multiple, indeed the epithelium of the entire oesophagus showed hyperplastic changes often involving primarily the basal-cell layer. Invasion of the muscle in the wall of the oesophagus was accepted as the criterion of malignancy. All the malignant tumours were squamous cell carcinomata. Not infrequently multiple malignant tumours were present. Distant metastases were not seen, perhaps because tumours, relatively early in their genesis, tended to interfere with the nutrition of animals. However, invasion of the thyroid gland was seen in a male rat treated with *N*-nitrosomethylaniline.

The oesophageal tumours seen in the nitrosoanabasine treated rats were very similar to those seen in groups 1 and 2. Low and high-power views of oesophageal tumours are shown in Fig. 3 and 4. One of the strange features of the results was the almost complete absence of abnormality in the squamous epithelium of the forestomach despite the presence of multiple tumours of the œsophagus. Slight epithelial hyperplasia was observed, but only in five rats, all of group 2, were tumours seen. In four of these the tumours were solitary small benign papillomas, and in one marked hyperplasia and benign papillomas were seen throughout the forestomach epithelium.

These data indicate clearly that nitrosoanabasine is carcinogenic though, in the doses given, slower in action than nitrosopiperidine and *N*-nitrosomethylaniline.

*Detection of nitrosamines*

Whilst the biological tests have been in progress attempts have been made to detect nitrosoanabasine and nitrosonornicotine in tobacco smoke. Methods for detecting these substances were developed. Both can be detected on either paper chromatograms or on thin-layer chromatograms. They can be reduced

---

EXPLANATION OF PLATE

FIG. 3.—Oesophagous, stomach and duodenum from a rat given nitrosopiperidine in the drinking water for a period of 206 days. The oesophagus is enlarged to many times its normal size by the presence of multiple benign and malignant squamous cell tumours.

FIG. 4.—Squamous carcinoma invading the muscular wall of the oesophagus from a rat given nitrosoanabasine in the drinking water for 419 days. H. and E. ×255.

3

4

with zinc and acetic acid to hydrazine derivatives, which give colours with *p*-dimethylaminocinnamaldehyde. These nitrosamines also react with an acidic solution of 2-(*N*-benzylanilinemethyl)imidazoline (Antistin) to give yellow colours after 20 minutes which gradually change to green-blue during 24 hours.

A more sensitive test in which the nitrosamines are decomposed in the presence of *p*-chloraniline—producing a diazonium compound which is then coupled with *N*-(1-naphthyl)ethylenediamine—has also been developed.

All these methods have been used in attempts to detect these substances in tobacco smoke, but so far without success. Other constituents of the smoke appear to interfere with the reactions, for even when nitrosoanabasine was injected into cigarettes before smoking, none was detected in the smoke. Moreover, nitrosoanabasine added to smoke condensate cannot be detected by these methods. Attempts to overcome these difficulties in detecting small amounts of nitrosoanabasine and other nitrosamines in cigarette smoke are being continued in the light of the positive result obtained.

### DISCUSSION

Nitrosoanabasine is one example of a nitrosamine which could be produced from an amine known to be present in tobacco smoke. Other amines present in tobacco smoke and whose corresponding nitroso derivatives are carcinogenic include dimethylamine, diethylamine, pyrollidine and piperidine, whilst the nitroso derivative of proline is practically inactive.

### SUMMARY

1. Nitrosoanabasine which could theoretically be formed in cigarette smoke and is a derivative of the known carcinogen nitrosopiperidine induced cancer of the oesophagus on administration to rats in drinking water for over 300 days.

2. *N*-Nitrosomethylaniline had similar carcinogenic activity to nitrosoanabasine but nitrosopiperidine was more active, inducing tumours of the liver and oesophagus so that all the treated animals were dead within 300 days.

3. Methods of detecting nitrosoanabasine and nitrosonornicotine were developed but neither of these nitrosamines could be detected in cigarette smoke. Failure to detect these nitrosamines might have resulted from their high chemical reactivity since they could not be detected in smoke from cigarettes to which they had been added, or in smoke condensates to which they had been added.

Miss Anne Walsh, Miss Ruth Dunkley and Mr. George Munro gave valuable technical assistance. We wish to thank the Tobacco Manufacturers' Standing Committee for defraying the cost of anabasine and nornicotine used in the synthesis of nitrosoanabasine and nitrosonornicotine used in these experiments. This investigation has been supported by grants to the Chester Beatty Research Institute (Institute of Cancer Research : Royal Cancer Hospital) from the Medical Research Council, the British Empire Cancer Campaign and the National Cancer Institute of the National Institutes of Health, U.S. Public Health Service.

### REFERENCES

BOKOVEN, C. AND NIESSEN, H. J.—(1961) *Nature, Lond.*, **192**, 458.

DRUCKREY, H., PREUSSMANN, R., SCHMAHL, D. AND MÜLLER, M.—(1961) *Naturwissenschaften*, **48**, 134.—(1962) *Ibid.*, **49**, 19.

Haagen-Smit, A. J., Brunelle, M. F. and Hara, J.—(1959) *Arch. industr. Hlth*, **20**, 399.
Magee, P. N. and Barnes, J. M.—(1956) *Brit. J. Cancer*, **10**, 114.—(1962) *J. Path. Bact.*, **84**, 19.
Quin, L. D.—(1959) *J. org. Chem.*, **24**, 914.
Roe, F. J. C., Salaman, M. H., Cohen, J. and Burgan, J. G.—(1959) *Brit. J. Cancer*, **13**, 623.

*Note added in proof:* Mice injected subcutaneously with nitrosonornicotine have developed multiple tumours of the lung showing that this nitrosamine is also carcinogenic.